# How the Brain Understands Spoken and Sung Sentences

**DOI:** 10.3390/brainsci10010036

**Published:** 2020-01-08

**Authors:** Sonja Rossi, Manfred F. Gugler, Markus Rungger, Oliver Galvan, Patrick G. Zorowka, Josef Seebacher

**Affiliations:** 1 ICONE-Innsbruck Cognitive Neuroscience, Department for Hearing, Speech, and Voice Disorders, Medical University of Innsbruck, 6020 Innsbruck, Austria; 2 Department for Medical Psychology, Medical University of Innsbruck, 6020 Innsbruck, Austria; manfred.gugler@tirol-kliniken.at; 3 Department for Hearing, Speech, and Voice Disorders, Medical University of Innsbruck, 6020 Innsbruck, Austria; markus.rungger@i-med.ac.at (M.R.); oliver.galvan@tirol-kliniken.at (O.G.); patrick.zorowka@i-med.ac.at (P.G.Z.); josef.seebacher@i-med.ac.at (J.S.)

**Keywords:** semantics, speech comprehension, singing, N400, event-related brain potentials (ERPs), functional near-infrared spectroscopy (fNIRS)

## Abstract

The present study investigates whether meaning is similarly extracted from spoken and sung sentences. For this purpose, subjects listened to semantically correct and incorrect sentences while performing a correctness judgement task. In order to examine underlying neural mechanisms, a multi-methodological approach was chosen combining two neuroscientific methods with behavioral data. In particular, fast dynamic changes reflected in the semantically associated N400 component of the electroencephalography (EEG) were simultaneously assessed with the topographically more fine-grained vascular signals acquired by the functional near-infrared spectroscopy (fNIRS). EEG results revealed a larger N400 for incorrect compared to correct sentences in both spoken and sung sentences. However, the N400 was delayed for sung sentences, potentially due to the longer sentence duration. fNIRS results revealed larger activations for spoken compared to sung sentences irrespective of semantic correctness at predominantly left-hemispheric areas, potentially suggesting a greater familiarity with spoken material. Furthermore, the fNIRS revealed a widespread activation for correct compared to incorrect sentences irrespective of modality, potentially indicating a successful processing of sentence meaning. The combined results indicate similar semantic processing in speech and song.

## 1. Introduction

Speech communication is a unique human ability. However, also listening to and playing music is only present in human people. Both speech and music include productive and perceptual aspects. In the present study, we will focus on perceptual abilities. Language as well as music processing can be partitioned into several sub-abilities such as the identification of single sounds, syntactic-combinatorial rule extraction, melodic perception, and meaning extraction [1]. We will put the emphasis on the perception of linguistic meaning in speech and song. Singing is a form of music which also carries direct semantic meaning as in spoken language but with additional melody. Speech and song differ with respect to several aspects: songs display a more precise articulation and longer vowel duration than speech [2,3]. Furthermore, pitch is altered in song exhibiting a more discrete F0 contour and a fine-grained accurate pitch processing compared to speech [4]. Singing is an important evolutionary phenomenon, as early humans already used a protolanguage similar to singing [5], and still nowadays the parent–children interaction is characterized by singing which supports bonding [6,7]. We opted for investigating this kind of music, as hearing impaired patients show more difficulties in extracting meaning from sung sentences (e.g., [8]). In a currently ongoing study in our lab, we aim at better understanding the neural processing of speech comprehension in different groups of hearing-impaired patients. We are particularly interested in whether they show similar or altered neural mechanisms to semantic processing. Before being able to interpret pathological data, it is however important to clearly understand processing mechanisms in healthy subjects. An overall musical training and/or singing in particular were found to positively impact semantic processing [9], foreign language learning in general [10,11], and perception of speech in noise [12,13,14]. Furthermore, music is beneficial for language production abilities during rehabilitation of language disorders such as aphasia [15,16,17,18,19]. Furthermore, deaf children supplied with a cochlear implant were found to benefit from musical training as they improve auditory abilities as well as language production and perception [20,21,22]. We are primarily interested in neural mechanisms as a direct measure of semantic processing and will compare these to behavioral performance during a correctness judgement task. Because different neuroscientific methods assess different neural signals of the brain, results may lead to different modulations and conclusions. Hence, we opt for a multi-methodological approach in which we simultaneously apply the electroencephalography (EEG) and the functional near-infrared spectroscopy (fNIRS). EEG, and—in particular—the investigation of event-related brain potentials (ERPs), assesses electrical signals from the scalp and bears the potential to assess fast dynamic processing mechanisms in the range of tens of milliseconds. The topographical resolution is only rough in EEG but in order to assess this information the fNIRS method represents an ideal candidate. fNIRS is an optical method assessing the vascular response by means of near-infrared light (for a review on fNIRS see [23]). Even though this response proceeds on a much larger timescale than EEG it allows for reliably identifying involved brain areas. It can measure brain responses from about 3 cm depth from the scalp. Through this, only cortical regions can be reached in adult participants. The combination of these two methods is perfectly suitable for investigation of auditory stimuli as (1) they are both soundless in contrast to the application of functional magnetic resonance imaging (fMRI), which produces loud noise during data acquisition; (2) they do not interfere with each other compared to EEG-fMRI; (3) they allow a comfortable measuring setting while subjects are seated in a chair instead of lying in an MRI scanner. 

### 1.1. Electrophysiological Correlates of Semantic Processing in Speech and Song

In language comprehension research, semantic processing was investigated through several experimental designs. One of these is the priming design. During this paradigm, a prime followed by a target stimulus is presented. Usually, the target stimulus is semantically related or unrelated to the preceding prime. An electrophysiological correlate of semantics elicited in priming paradigms is the N400 component. The amplitude of the N400 reduces with repetition of stimuli and was thus found to be enhanced for unrelated targets (e.g., [24,25,26,27]). This centro-parietal ERP component reflects the degree of semantic relatedness between prime and target, and is thus an index of semantic processing (for a review see [28]). A similar paradigm was also adopted for investigating meaning in music. Some studies addressed the question whether instrumental music without lyrics can convey extra-musical meaning such as iconic, indexical/emotional, or symbolic/cultural meaning or intra-musical meaning (i.e., structure of musical elements) (please refer to reviews by [29,30]). As primes, musical excerpts without lyrics [31], single chords [32], or single tones [33] were used followed by a semantically related or unrelated target word. A similar N400 modulation (i.e., larger amplitude for unrelated targets) as for speech was also found in this musical context suggesting shared mechanisms of semantic priming in speech and music. Electrophysiological studies specifically investigating sung material are scarce. However, semantic processing in songs also elicited larger N400s for target words unrelated to the final word of familiar and unfamiliar pop song excerpts compared to related words [34] or when sung target words differed from sung prime words compared to the repetition of the same words [35]. 

Another important paradigm suitable for investigating perception of semantic processing is to integrate selection restriction errors in sentences and compare them to semantically correct sentences [36,37]. Such a design can be adopted in both spoken and sung sentences or phrases. Similar to the semantic priming paradigm, also in this experimental context, the N400 component in the EEG reliably indexes the integration of semantic information. The amplitude was found to be larger for semantically incorrect compared to correct sentences [36,38,39]. To our knowledge, there is only one electrophysiological study integrating semantic errors in sung musical excerpts [40]. In this study, familiar excerpts from French operas which were sung a cappella were presented to professional opera musicians. Songs were manipulated in such a way that the original version was contrasted with a version containing either semantically incorrect final words or melodic incongruities. Semantically incorrect words elicited a larger N400 component compared to correct words in these sung excerpts. This finding is in line with a magnetoencephalographic study in professional singers and actors using spoken and sung excerpts from Franz Schubert in which, above all, the final word was either semantically correct or incorrect [41]. 

Electrophysiological findings seem to suggest that semantic processing in speech and song is supported by similar processing mechanisms (for a review please refer to [42]). However, there is no study so far which directly compares electrophysiological processes in relation to selection restriction errors in spoken and sung sentences.

### 1.2. Brain Regions Supporting Semantic Processing in Speech and Song

Several models tried to assign different aspects of speech and music to the two hemispheres of the brain. Zatorre and colleagues postulated that auditory cortices of both hemispheres are specialized for different auditory analyses whereby the left temporal area is more sensitive for fine-grained temporal analyses and the right temporal area reacts more to spectral variations [43,44]. This difference led to the conclusion that speech is processed predominantly by left and music by right temporal areas [43]. The multi-time resolution hypothesis proposed by Poeppel and colleagues postulates a dichotomy of the left and right auditory cortices based on temporal variations contained in speech and music [45]. Fast auditory transitions are assumed to be processed bilaterally while slow transitions predominantly recruit right temporal areas. Such a hemispheric specialization is already visible in newborn infants when confronted with auditory stimuli with varying temporal modulations [46]. These models predominantly focus on the auditory cortex. The Dynamic Dual Pathway model [47], in contrast, differentiates between different linguistic functions and allocates them to cortical regions of the two hemispheres. The model postulates that segmental information such as phonology, syntax, and semantics are predominantly processed by a fronto-temporal network in the left hemisphere while prosody is located primarily in homologous right-hemispheric areas.

When focusing on semantic processing in particular, a ventral stream including the superior and middle portions of the temporal lobe was proposed [48]. This stream seems to be bilaterally distributed with a weak left-hemispheric dominance. A more or less dominant lateralization usually depends on the linguistic or musical aspects contrasted with each other.

Brain regions activated by priming paradigms—and thus supporting semantic relatedness, access to the lexical storage, and semantic selection—were found to be located predominantly in temporal (particularly the middle temporal gyrus (MTG) and the superior temporal sulcus (STS)), and frontal regions (especially the inferior frontal gyrus (IFG) and orbitofrontal cortex (OFC)) in speech (e.g., [49,50,51]) but also in music [32]. Using unfamiliar songs which were repeated either with the same or different lyrics or with the same or different tunes, non-musicians showed a larger left-hemispheric activation in anterior STS for lyrics in contrast to tunes suggesting a greater autonomy of linguistic meaning probably because participants could rely more on their linguistic than musical expertise [52]. It should be noted that this study did not introduce any experimental task, thus subjects simply passively listened to the same/different repetition. In contrast, Schön and colleagues [53] presented pairs of spoken, vocalized (i.e., sung without words), or sung words and asked subjects to explicitly judge whether word pairs were the same or different. The authors found similar brain regions being activated in spoken, vocalized, and sung processing compared to a noise stimulus. Differences arose at a quantitative rather that qualitative level. A larger activation in the left IFG was found for sung compared to vocalized words as they contained real words, thus more linguistically relevant features. Temporal areas (MTG and the superior temporal gyrus (STG)), on the contrary, were found for both linguistic and non-linguistic features, leading to the conclusion that these regions are recruited domain-independently.

Some neuroimaging studies compared different degrees of melodic information together with speech/lyrics and found differences especially in hemispheric lateralization. Merrill and colleagues [54] presented six different sentence categories: spoken, sung, with hummed (i.e., including prosodic pitch) or song melody (i.e., including melodic pitch), and with speech or musical rhythm. While activations were similar in bilateral temporal lobes, differences were present with respect to the inferior frontal areas. Sung sentences elicited increased activations in the right IFG similar to melodic pitch. Prosodic pitch, on the contrary, gave rise to activations predominantly in the left IFG. These findings fit with results obtained in a study [55] contrasting linguistic prosody (i.e., whether the phrase was spoken as a statement or question) to speech recognition (i.e., identifying whether a word was the same or different relative to the previous word). In this contrast, a larger recruitment of a right-hemispheric temporo-frontal network was found for linguistic prosody because of a stronger reliance on prosodic, thus melodic, aspects. The degree of linguistic or melodic features contained in the presented acoustic material seems relevant for a correct interpretation of found activations. In this vein of reasoning, a direct comparison between the listening to/production of spoken and sung material (e.g., familiar songs, words, phrases) showed an increased right-hemispheric dominance of the middle STG, the planum temporale (PT), and the OFC for sung compared to spoken songs [56,57,58]. The authors interpret the PT to be involved in the transformation of auditory input into motor representation relevant for speech production. The OFC is assumed to process pleasant and unpleasant emotional aspects during music perception. Interestingly, when comparing sung (i.e., linguistic and melodic information) as well as instrumental music (i.e., only melodic information) to spoken songs (i.e., only linguistic information), an increased activation was found not only in the right planum temporale but also in bilateral anterior planum polare, suggesting that these regions encode music/timbre in both instrumental and sung music [57]. Furthermore, spoken and sung material activated the STS bilaterally, indicating that this area is sensitive to human nonlinguistic vocalizations. 

Brain regions subserving semantic processing at the sentential level in speech similarly include activations in left or bilateral temporal (particularly in STG and MTG), left or right frontal areas, and sometimes left parietal areas (i.e., angular gyrus) [59,60,61,62]. Bilateral temporal areas are assumed to reflect the semantic integration or semantic evaluation of the sentence, however some fMRI studies found increased activations in this region for correct compared to incorrect sentences [59] while others revealed a reversed activation pattern [62]. Frontal regions were found to be associated with semantic selection processes [61,62] whereas left temporal and temporo-parietal areas were also discussed to be involved in the integration of different syntactic and semantic information in a sentence [59,63]. While such a paradigm which integrates semantic errors in sentences was successfully applied in language research, to date no neuroimaging study used this paradigm for investigating semantic processing in songs. In the present study we opted for integrating semantic anomalies in sentences which were either spoken or sung in order to directly compare the underlying neural processing mechanisms.

### 1.3. The Present Study

The focus of the present study lies on semantic processing in speech and song. Even though several studies examined semantics by means of a priming design, it is not well understood how melodic aspects contribute to the extraction of meaning from semantically correct and incorrect sentences. Thus, we created a set of semantically correct and incorrect sentences which were either spoken or sung. While subjects listened to these sentences and performed a correctness judgement task, neural processing was assessed via the simultaneous application of the electroencephalography (EEG) and the functional near-infrared spectroscopy (fNIRS). This multi-methodological approach was chosen for several reasons: (1) only one electrophysiological study [40] so far investigated semantic errors in sung sentences in professional musicians, but no direct comparison with spoken sentences in the same non-musically trained subjects was performed until now, (2) no neuroimaging study so far directly investigated semantic errors in sung sentences, and (3) the use of fNIRS in contrast to fMRI, especially, is very advantageous as this method is completely silent without any scanner noise, and is thus suitable for measuring acoustic stimuli. In the EEG we will focus on the well-established ERP component of the N400, while the fNIRS is capable of identifying underlying brain areas. In particular, the involvement of same or different neural networks in sung and spoken sentences as well as the degree of lateralization will provide important insights into the neural underpinnings of semantic processing in speech and song and potentially be relevant for therapeutic interventions in hearing impaired patients in future. 

## 2. Materials and Methods

### 2.1. Participants

Twenty German native speakers (10 female) participated in the study (mean age: 38.65 years; range: 28–53 years). All participants grew up monolingually with German, and had learned foreign languages mostly at school or through friends, but not intensively in their family surroundings like bilingual subjects. All subjects learned English as their first foreign language at a mean age of 10.67 years (range: 3–11 years; 1 missing data). Other foreign languages were also learned (1 subject had learned 4 additional foreign languages, 2 subjects had learned 3 additional foreign languages, 4 subjects had learned 2 additional foreign languages, 7 subjects had learned 1 additional foreign language). All subjects were right-handed according to the Oldfield Handedness Inventory [64] (mean % right-handedness: 73.68; range: 0–100), had no neurological disorders, were not born prematurely, took no medication affecting cognitive functioning, had normal or corrected-to-normal vision and a normal hearing ability at both ears (assessed by an otorhinolaryngologist and an audiologist of the Department of Hearing, Speech, and Voice Disorders of the Medical University of Innsbruck by means of a pure tone audiogram (PTA) with the following criteria: thresholds <30 dB HL at audiometric test frequencies 500, 1000, 2000, and 4000 Hz-PTA4 average). No subject was a professional musician. 18 subjects entered EEG analyses (2 were excluded due to technical problems) and 18 subjects entered fNIRS analyses (2 were excluded due to technical problems). The excluded subjects differed between EEG and fNIRS as technical problems only referred to one single method. The respective other method remained unaffected.

### 2.2. Materials

The language material consisted of 88 German sentences (44 semantically correct, 44 semantically incorrect) of the following structure: definite article-subject-auxiliary-definite article-object-past participle). All sentences were constructed in past perfect tense. All nouns (subject and object) were bisyllabic, past participle verbs were trisyllabic. Example of a correct sentence: “Der Forscher hat die Firma gegründet” (engl. translation with German word order: “The researcher has the company founded”). Example of an incorrect sentence: “Der Forscher hat die Birke gegründet” (engl. translation with German word order: “The researcher has the birch founded”). Semantic incorrectness was achieved by a selection restriction error. 

All correct and incorrect sentences were naturally spoken and sung by a male speaker who was working as a speech therapist and was trained as a professional singer. Sung sentences were assigned to four different melodies (2 rising, 2 descending) whereas rhythm was kept constant in order to provide a greater melodic variety to subjects (please refer to Supplementary Materials for an auditory example of a correct/incorrect spoken/sung sentence). Acoustic stimuli were digitally recorded in an anechoic chamber at a sampling rate of 44 kHz and 16 bits. Afterwards, acoustic stimuli were edited using the editing program Audacity (www.audacityteam.org). This included inserting 30 ms of silence at the onset and offset of each sentence as well as loudness normalizing. Furthermore, each individual word of the sentences was marked, and the individual onset times of each word were extracted. This was necessary in order to insert the exact timing of each word into the EEG and fNIRS marker files for neuroscientific analyses. Duration of the critical verb was as following: correct spoken: 1198 ms, incorrect spoken: 1190 ms, correct sung: 1744 ms, and incorrect sung: 1722 ms. An ANOVA with the within-subject factors *condition* (correct vs. incorrect) and *modality* (spoken vs. sung) revealed a significant main effect of *modality* [*F* (1,43) = 449.293, *p* < 0.0001] suggesting longer verbs in sung compared to spoken sentences. We also tested the whole duration of sentences and found a similar main effect of *modality* [*F* (1,43) = 130.862, *p* < 0.0001]. Again, sung sentences were longer than spoken ones (correct spoken: 4492 ms, incorrect spoken: 4362 ms, correct sung: 5193 ms, and incorrect sung: 5069 ms). 

### 2.3. Experimental Procedure

The present study was approved by the Ethics Committee of the Medical University of Innsbruck (approval code: 1041/2017). Prior to participating in the experiment, subjects were informed in detail about the aims of the study, the sequence of the experiment, the methods, the exact application procedures, the risks, and the actions to minimize these risks. After having the possibility to clarify any questions, subjects gave written informed consent to take part in the study. Subjects did not receive any compensation for participation.

The experiment was controlled by means of the software Presentation (www.neurobs.com). The presentation sequence started with a fixation cross for 500 ms on a 24‘’ monitor positioned 1 m in front of the subject. Afterwards the acoustic presentation of the sentence started via stereo loudspeakers positioned below the monitor. Sentences were presented at a sound level of approximately 70 dB. The maximum duration of a slot to present a sentence was 6 s. During this time the fixation cross remained on the screen in order to mitigate effects of eye movements on the EEG signal. After the sentence the fixation cross was again presented for 500 ms. This was followed by the visual presentation of a sad and a happy smiley initiating the correctness judgement task. During this task, subjects had to press either the left or right mouse button indicating whether the previously heard sentence was semantically correct (indicated by a happy smiley) or not (indicated by a sad smiley). The position of the smileys on the monitor as well as the required button presses was counter-balanced across participants. Subjects had to respond within 3 s and the presentation sequence continued as soon as they pressed the button. Afterwards a variable inter-stimulus-interval (ISI) of 6 s on average (range: 4–8 s) followed. This long ISI had to be introduced because of the assessment of functional near-infrared spectroscopy which measures the sluggish hemodynamic response (HRF) peaking around 5 s and returning to baseline after 15–20 s [65]. Because the HRF for each sentence would overlap in time, the introduction of a variable ISI prevents a systematic overlap and allows disentangling brain activation for each experimental condition. 

Eight different pseudo-randomization versions were created based on the following rules: (1) not more than 3 correct or incorrect sentences in succession, (2) not more than 3 spoken or sung sentences in succession, (3) at least 10 items between sentences of the same sentence pair, (4) in each experimental half an equal amount of correct and incorrect sentences, and (5) in each experimental half the same amount of spoken and sung sentences. 

Completing the experiment took about 45 min on average for all participants. In order to prevent subjects’ fatigue, two standardized pauses were introduced after each 15 min. 

### 2.4. Neuroscientific Recording

#### 2.4.1. EEG Recording

**Figure 1:**
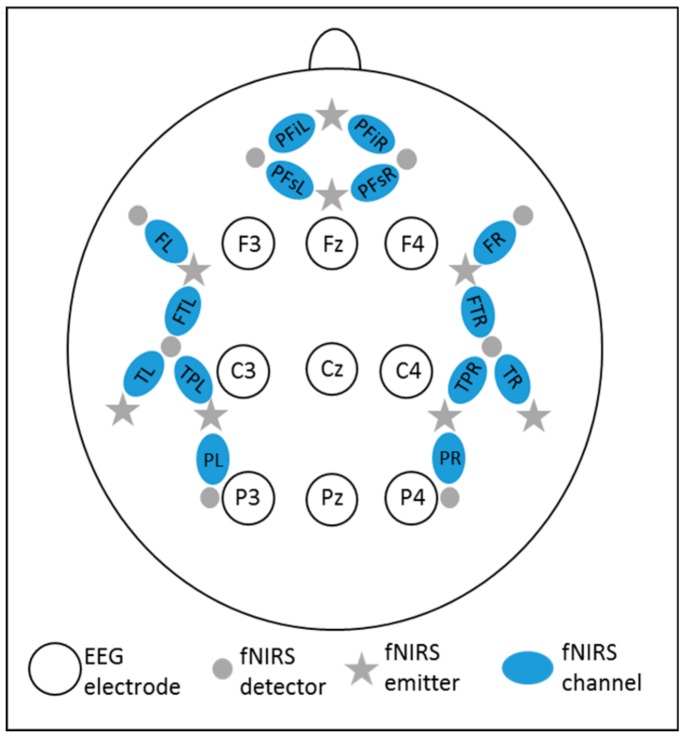
EEG-fNIRS positioning. PFi = prefrontal inferior, PFs = prefrontal superior, F = frontal, FT = fronto-temporal, T = temporal, TP = temporo-parietal, P = parietal, L = left, R = right.

The electroencephalogram (EEG) was recorded from 13 AgAgCl active electrodes (Brain Products GmbH, Gilching, Germany). Nine electrodes were placed on the scalp (F3, Fz, F4, C3, Cz, C4, P3, Pz, P4; see Figure 1), while the ground electrode was positioned at AFz and the reference electrode at the nasal bone. One electrode above the right eye (at position FP2) measured the vertical electro-oculogram while one electrode at the outer canthus of the right eye (at position F10) assessed the horizontal electro-oculogram. Electrode impedance was controlled using actiCap Control software (Brain Products GmbH, Gilching, Germany) and kept below 10 kΩ. The EEG signal was recorded by means of the software Brain Vision Recorder (Brain Products GmbH, Gilching, Germany) at a sampling rate of 1000 Hz and amplified between 0.016 and 450 Hz. An anti-aliasing filter with a cut-off at 450 Hz (slope: 24 dB/oct) was applied prior to analogue to digital conversion.

#### 2.4.2. fNIRS Recording

Functional near-infrared spectroscopy (fNIRS) was recorded by means of the NIRScout device (NIRx Medical Technologies, LLC, USA), using a dual wavelength (850 and 760 nm) continuous-wave system. Signals were recorded from 14 channels, resulting from a combination of 8 light emitters and 8 light detectors positioned over bilateral prefrontal, frontal, temporal, temporo-parietal, and parietal areas (see Figure 1). The emitter–detector distance was 3.5 cm. Sampling rate was 7.81 Hz.

### 2.5. Data Analyses

#### 2.5.1. Behavioral Data Analyses

Based on the correctness judgement task subjects had to indicate whether the heard sentence was semantically correct or incorrect. Percentage of correct responses as well as associated reaction times were extracted and analyzed by means of an ANOVA with the within-subject factors *condition* (correct vs. incorrect) and *modality* (spoken vs. sung). Significance level was set at *p* < 0.05. In case of a significant interaction, post-hoc *t*-tests were performed adjusted by the False Discovery Rate [66].

#### 2.5.2. EEG Data Analyses

EEG data analyses were performed with the software Brain Vision Analyzer 2 (Brain Products GmbH, Gilching, Germany). EEG data were first low-pass filtered with a cut-off of 30 Hz (slope: 12 dB/oct, Butterworth zero-phase filter). Afterwards, a segmentation based on the critical verb in the sentence was performed from 200 ms before verb onset until 1500 ms after verb onset. An ocular correction based on the Gratton and Coles algorithm [67] was applied in order to correct vertical eye blinks. Other artifacts were manually rejected. A baseline correction (−200–0 ms) was applied. Event-related brain potentials (ERPs) were extracted for each subject and each experimental condition (correct spoken, incorrect spoken, correct sung, incorrect sung) which was followed by the calculation of grand averages in a time window from −200 ms until 1500 ms time-locked to verb onset. After artifact rejection 75.6% (range: 50%–96.2%) of correct spoken, 75% (range: 39.4%–97%) of incorrect spoken, 76.6% (range: 51.3%–94.4%) of correct sung, and 77.8% (range: 51%–95.2%) of incorrect sung sentences entered final statistical analyses.

Statistical analyses were conducted on mean amplitudes. Because the difference between correct and incorrect sentences for spoken and sung sentences was delayed in time, two time windows, 500–900 ms and 800–1200 ms, were chosen based on visual inspection of the grand averages. The first time window characterized the N400 differences between correct and incorrect sentences for spoken sentences, while the second time window indicated the difference for sung sentences. For these analyses, a repeated-measures ANOVA with the within-subject factors *condition* (correct vs. incorrect), *modality* (spoken vs. sung), *region* (anterior vs. central vs. posterior), and *hemisphere* (left vs. right) was performed for lateral electrodes. Midline electrodes underwent an ANOVA with the factors *condition*, *modality*, and *electrodes*. With respect to modality, the mean amplitudes of the two time windows were used in the above-mentioned statistical analysis. Significance level was set at *p* < 0.05. Posthoc *t*-tests were performed and the False Discovery Rate [66] was applied for correcting for multiple comparisons. Whenever Mauchly’s test of sphericity became significant, the Greenhouse–Geisser correction [68] was applied. 

#### 2.5.3. fNIRS Data Analyses

fNIRS data were first separated into artifact-free segments by eliminating potential artifact-contaminated segments at the beginning and end of experiment as well as additionally introduced pauses in between the experiment in which no markers were presented. Further artifacts during the experiment were visually selected and corrected by a linear interpolation approach (e.g., [69]). A low-pass Butterworth filter of 0.4 Hz (filter order: 3) was applied. Stimulus duration was set at 3 s and used afterwards for applying the general linear model (GLM). Light attenuation was converted into concentration changes of oxygenated hemoglobin [oxy-Hb] and deoxygenated hemoglobin [deoxy-Hb] by means of the modified Beer–Lambert law [70]. For statistical analyses, a GLM-approach was used—in which a box-car-predictor of the stimulus duration was convolved with a canonical hemodynamic response function [71]—peaking at 5 s and fitted to the measured data. This procedure resulted in Beta-values corresponding to µmolar changes which were used for statistical analyses. These comprised repeated-measure ANOVAs with the within-subject factors *condition* (correct vs. incorrect), *modality* (spoken vs. sung), *region* (each of the 7 channels), and *hemisphere* (left vs. right), performed for [oxy-Hb] and [deoxy-Hb], separately. The significance level was set at *p* < 0.05. Posthoc *t*-Tests were performed and the False Discovery Rate [66] was applied for correcting multiple comparisons. Whenever Mauchly’s test of sphericity became significant Greenhouse–Geisser correction [68] was applied. Increases in [oxy-Hb] as well as decreases in [deoxy-Hb] are both signs of increased brain activation and were thus analyzed separately.

## 3. Results

### 3.1. Behavioral Results

**Figure 2:**
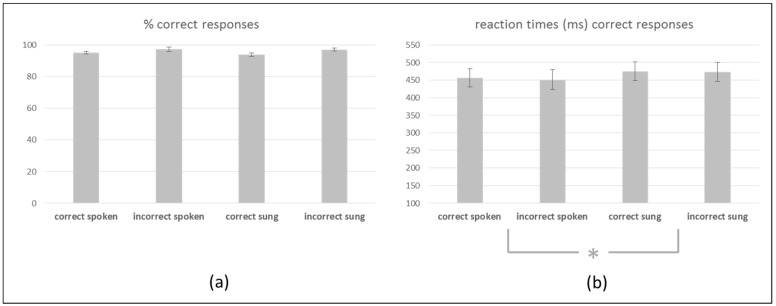
Behavioral data from the correctness judgement task. (**a**) Percentage of correctly answered trials per experimental condition including SEMs. (**b**) Reaction times (in ms) of correctly answered trials per experimental conditions including SEMs. * indicates the significant main effect of *modality* reflecting longer reaction times for sung compared to spoken sentences.

The ANOVA with respect to percentage of correctly answered trials during the correctness judgement task yielded no significant main effect or interaction indicating an equally high percentage: correct spoken (95%), incorrect spoken (97%), correct sung (94%), and incorrect sung (97%) (please refer to Figure 2).

The ANOVA for reaction times of correctly answered trials during the correctness judgement task yielded a significant main effect of *modality* [*F* (1,19) = 4.602, *p* = 0.045]. Posthoc *t*-tests revealed longer reaction times for sung (474 ms) compared to spoken sentences (454 ms) (see Figure 2).

### 3.2. EEG Results

**Figure 3:**
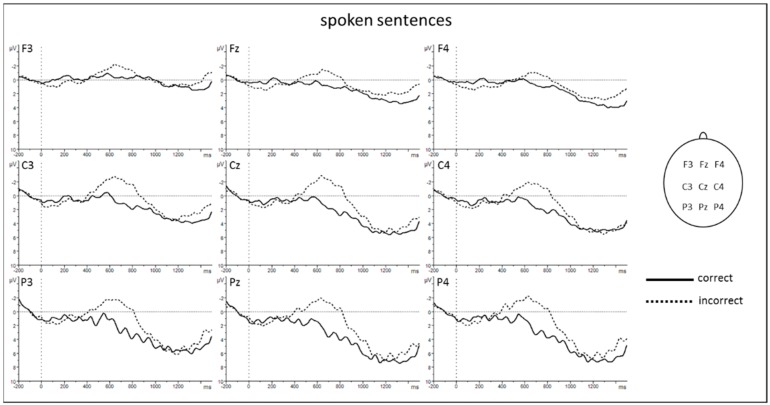
ERP results for spoken sentences. Grand averages from −200 ms to 1500 ms after verb onset. Negativity is plotted upwards. An 8 Hz low-pass filter was applied for presentation purposes only.

**Figure 4:**
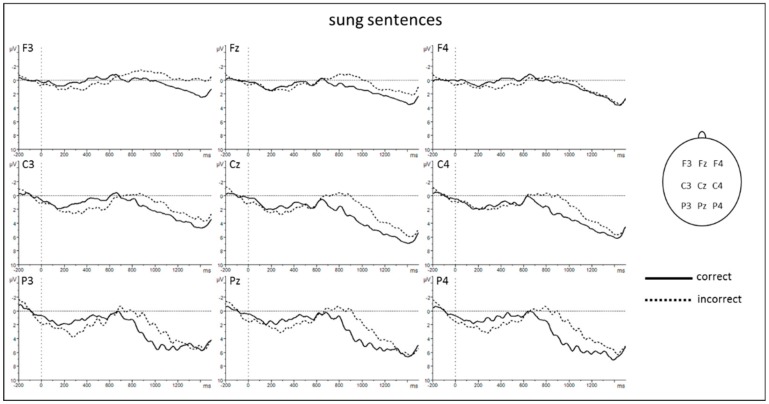
ERP results for sung sentences. Grand averages from −200 ms to 1500 ms after verb onset. Negativity is plotted upwards. An 8 Hz low-pass filter was applied for presentation purposes only.

The ANOVA for lateral electrodes revealed significant main effects of *condition* and *modality* as well as significant interactions *condition* × *region* and *modality* × *region* (Table 1). Subsequent posthoc *t*-Tests resolving the interaction *condition* × *region* revealed a larger negative amplitude for incorrect compared to correct sentences at central [C3 and C4: *t* (17) = 3.326, *p* = 0.004] and posterior regions [P3 and P4: *t* (17) = 3.223, *p* = 0.005] (see Figure 3 and Figure 4). Posthoc *t*-tests resolving the interaction *modality* × *region* revealed a larger negativity for spoken compared to sung sentences at central [C3 and C4: *t* (17) = −3.064, *p* = 0.007] and posterior regions [P3 and P4: *t* (17) = −3.076, *p* = 0.007]. 

Findings for midline electrodes revealed the following significant effects (Table 2): main effect of *condition*, main effect of *modality*, and interaction *condition* × *electrodes*. The main effect of modality revealed a more negative shift for spoken compared to sung sentences. Subsequent posthoc *t*-tests resolving the interaction *condition* × *electrodes* revealed a larger negativity for incorrect compared to correct sentences at Fz [*t* (16) = 3.199, *p* = 0.006], Cz [*t* (17) = 3.340, *p* = 0.004], and Pz [*t* (17) = 3.264, *p* = 0.005] (see Figure 3 and Figure 4).

### 3.3. fNIRS Results

#### 3.3.1. Results for [oxy-Hb]

**Figure 5:**
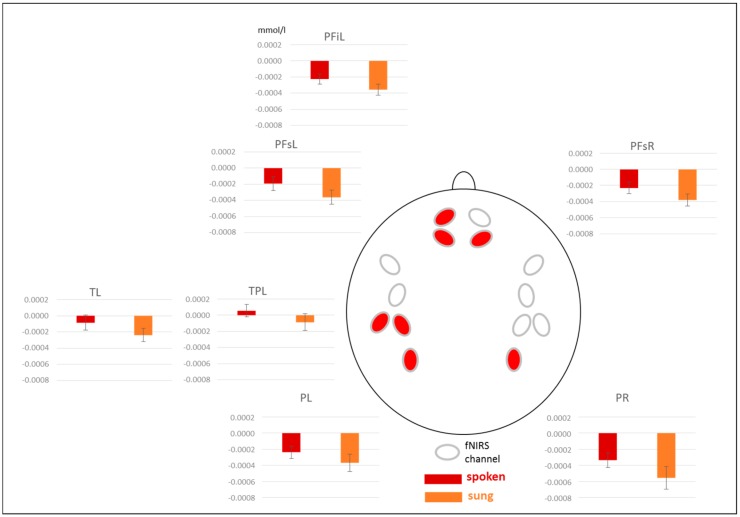
fNIRS results for [oxy-Hb]. Beta-values for spoken and sung sentences merged across correct and incorrect sentences. Red channels indicate significant differences. PFi = prefrontal inferior, PFs = prefrontal superior, T = temporal, TP = temporo-parietal, P = parietal, L = left, R = right. Please note that a more positive value indicates an increased activation.

The ANOVA revealed a significant main effect of *modality* as well as significant interactions *modality* × *region* and *modality* × *region* × *hemisphere* (Table 3). Subsequent posthoc *t*-tests resolving the three-way interaction revealed a stronger activation for spoken compared to sung sentences at the following channels: left prefrontal inferior [PFiL: *t* (17) = 2.974, *p* = 0.009], left and right prefrontal superior [PFsL: *t* (17) = 2.615, *p* = 0.018; PFsR: *t* (17) = 2.814, *p* = 0.012], left temporal [TL: *t* (17) = 2.140, *p* = 0.047], left temporo-parietal [TPL: *t* (17) = 2.902, *p* = 0.010], as well as left and right parietal [PL: *t* (17) = 2.242, *p* = 0.039; PR: *t* (17) = 3.041, *p* = 0.007] (see Figure 5). 

#### 3.3.2. Results for [deoxy-Hb]

**Figure 6:**
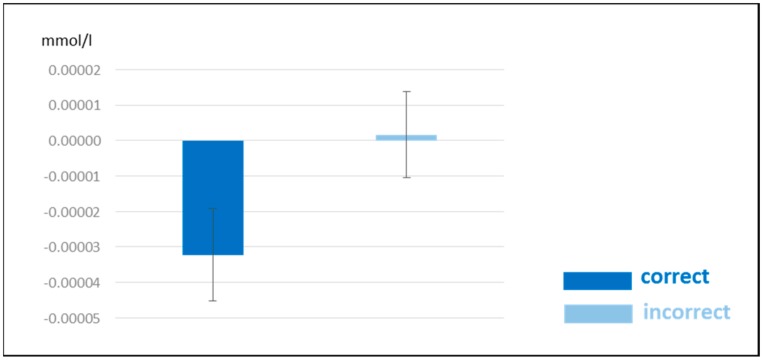
fNIRS results for [deoxy-Hb]. Beta-values for correct and incorrect sentences merged across spoken and sung sentences and across all channels including SEMs. Please note that a more negative value indicates an increased activation.

The ANOVA revealed a significant main effect of *condition* (Table 4) indicating a stronger activation for correct compared to incorrect sentences (see Figure 6). 

## 4. Discussion

The present study investigated neural mechanisms of semantic processing in speech and song. Semantic processing was operationalized by acoustically presenting semantically correct and incorrect sentences which were either spoken or sung. Singing is a form of music including both melodic as well as linguistic aspects. However, is meaning extracted similarly or differently from singing compared to pure spoken information? This research question guided the present study. In order to assess neural foundations of semantic processing, two neuroscientific methods were applied simultaneously, namely the EEG and the fNIRS. 

### 4.1. The N400 Differentiates between Correct and Incorrect Sentences

EEG results for spoken and sung sentences showed a clear difference between semantically correct and incorrect sentences indexed by a classical N400 component. The N400 is usually found in several semantic contexts and reflects lexical access and semantic integration [27,28,37]. It shows larger amplitudes when semantic processing is difficult. Such a modulation was also found in our study, revealing larger N400 amplitudes for incorrect compared to correct sentences. This N400 effect was equally present in both modalities. However, an important difference was nevertheless observable. The N400 for spoken and sung sentences was generally delayed compared to previous studies, and the N400 for sung sentences was even more delayed (500–900 ms for spoken and 800–1200 ms for sung sentences). A first consideration for this general delay of the N400 was that the critical verb is a past participle containing a clear syntactic marker “ge” in German. Only after this prefix an identification of semantic correctness is possible. Thus, we averaged ERPs aligned after this prefix. However, the N400 for sung sentences was still delayed in time compared to spoken sentences (cf. Supplementary Figure S5). Thus, we opted for carrying out the standard analysis procedure aligning ERPs to critical word onsets. Another explanation for the delayed N400 might concern the subjects’ age range (mean age of 39 years). Studies investigating the N400 in differential semantic paradigms in younger (usually in the mid 20s) and older subjects show ambiguous results. Some studies report some delays of the N400 in older subjects [72,73,74] while others do not find any delayed processing [40,75,76]. Our delay might rather be driven by the longer duration of spoken but especially sung sentences as well as final words. A classical N400 to spoken sentences was usually reported between 300 and 500 ms [28]. In our study, the N400 to spoken sentences was found between 500 and 900 ms, thus delayed. However, giving a closer look to the grand averages of N400s in spoken sentences in previous studies shows that even though smaller time windows were analyzed (400–700 ms in [38] and 250–700 ms in [39]), the differences between semantically correct and incorrect sentences lasted longer (~until 1000 ms). This was the case for young (around 25 years [38,39]) but also middle age (around 43 years [77]) and older participants (around 60 years [78,79]). It should be noted that sentence duration in these studies [38,39] was about 1700 ms while in our study spoken sentences lasted much longer (around 4400 ms). This longer duration resulted from a slow presentation rate in order to approximate sentence length of spoken to sung sentences. Furthermore, this slow presentation rate was introduced because the study is currently also performed in hearing impaired patients supplied with cochlear implants and/or hearing aids with difficulties in language comprehension. In order to give these patients a chance to understand these sentences they were spoken very slowly. In fact, normal-hearing participants noticed this slow presentation rate, indicating that they experienced the experiment as effortful. Patients, on the other hand, did not complain about this slow presentation rate. Unfortunately, Besson and colleagues [40] do not report the exact duration of their sung final word. Gordon and colleagues [35], however, report the duration of their word stimuli used in a priming study. Their sung stimuli were 913 ms long while our critical words lasted around 1700 ms, thus much longer. While in Gordon et al. the N400 occurred between 300 and 500 ms, the longer duration of the sung stimuli in our study could explain the delayed N400 effect. Further support for this assumption is provided by the reaction times during the correctness judgement task in the present experiment also showing longer reaction times for sung compared to spoken stimuli. Finally, EEG results seem to show qualitatively similar semantic processing in spoken and sung sentences, with a quantitative difference displayed in a delayed N400 component. These EEG findings might be important with respect to hearing impaired patients who clearly show more behavioral difficulties in extracting meaning from sung sentences as from spoken speech [8] but also benefits from a musical training [21,22]. These findings are moreover interesting in the light of therapeutic interventions such as melodic intonation therapy (MIT) postulating a beneficial effect on language processing in aphasic patients through singing [15,16]. It should, however, be considered that MIT predominantly reveals its favorable effects with respect to speech production and not necessarily speech comprehension which was studied in the present study. 

### 4.2. Brain Areas Recruited for Semantic Processing in Spoken and Sung Sentences

fNIRS results showed a twofold pattern: (1) an increased activation for spoken compared to sung sentences, irrespective of semantic correctness in bilateral prefrontal, left temporal and temporo-parietal, and bilateral parietal areas, and (2) an increased activation for correct compared to incorrect sentences—irrespective of modality widespread over the whole cortex.

The larger activation for spoken compared to sung sentences in the fNIRS goes in line with the larger negativity for spoken versus sung sentences in the EEG. However, in the EEG this difference can hardly be interpreted due to the different time windows analyzed for spoken and sung sentences. This increased activation for spoken compared to sung sentences in the fNIRS shows a stronger left-hemispheric lateralization, which might potentially be driven by the fact that our participants were non-musicians. Thus, they are more familiar with understanding spoken compared to sung language in everyday life. Furthermore, the correctness judgement task directed attention to the linguistic content and not to the melodic features of sentences. Similar findings were also shown by Sammler and colleagues [52] in a repetition priming study with fMRI contrasting lyrics and tunes in unfamiliar songs. The authors also found larger activations in the left superior temporal sulcus for lyrics than tunes in musically untrained subjects suggesting a link between subjects’ expertise with music and language and a predominant processing of linguistic meaning. 

The second important fNIRS finding was the widespread increased activation for correct compared to incorrect sentences, irrespective of modality. This result is in line with previous studies which also contrast semantically correct to incorrect sentences [59,60,61]. In particular, the direction of effects conforms to the fMRI findings of Humphries and colleagues [59]. They contrasted semantically correct with random sentences (i.e., words were scrambled resulting in a meaningless sentence). The authors also found increased activations for correct sentences in similar regions as in our study. Especially temporo-parietal areas were proposed to be related to combinatory semantic processes at the sentence level relevant for the formation of a more complex meaning. Such an interpretation would also fit with our activation pattern. The fact that a differentiation between correct and incorrect sentences was equally present for spoken and sung material might be attributed to the task in our experiment which primarily directed attention to the semantic content of sentences. 

In general, however, topographic aspects of fNIRS results should be considered with caution as spatial resolution is limited compared to fMRI due to the possibility to assess neural activation from maximally 3 cm depth from scalp. Thus, only cortical areas can be reached. Due to the simultaneous assessment of EEG and fNIRS, only a limited number of light emitters and detectors can be positioned in between EEG electrodes. Consequently, specific tomographic analyses with multi-distance emitter-detector-pairs potentially leading to a better spatial resolution are not possible.

## 5. Conclusions

Findings from our multi-methodological approach indicate that the extraction of meaning from sentences is equally processed in spoken compared to sung sentences. A predominant processing of spoken compared to sung sentences could furthermore be attested. This effect seems to be at least partially influenced by a stronger familiarity with spoken material as well as with the correctness judgement task directing subjects’ attention to the linguistic content of sentences. It would be interesting to conduct the same experiment without any experimental task; for example, simply during passive listening to spoken and sung sentences. Importantly, these fine-grained mechanisms appear only in the neural response but not in behavioral data, showing an equally high percentage of identification of correct and incorrect sentences in both spoken and sung modality. Interestingly, both neuroscientific methods show concordant results with respect to the direction of effects. However, the EEG—with its high temporal resolution—showed quantitative differences between spoken and sung sentences, as semantic processing in sung sentences was delayed in time. Based on these findings, we pursue the next step to investigate semantics in spoken and sung sentences in hearing-impaired listeners who are supplied with either hearing aids or cochlear implants, as these patients experience language comprehension problems. This would provide insights into the neural processing mechanisms which are present at the beginning and during the course of the rehabilitation process. 

## Figures and Tables

**Table 1:** Statistical results of the ANOVA *condition* × *modality* × *region* × *hemisphere* for event-related brain potentials (ERP) data on lateral electrodes. Data from the time window 500–900 ms was considered for spoken sentences, while data from the time window 800–1200 ms entered analyses for sung sentences. Significant effects (*p* < 0.050) are marked in bold.

*Effect Lateral Electrodes*	*df*	*F*	*p*
***condition***	1,17	8.498	**0.010**
***modality***	1,17	8.572	**0.009**
*condition* × *modality*	1,17	0.221	0.644
***condition* × *region***	2,34	6.316	**0.010**
***modality* × *region***	2,34	6.929	**0.010**
*condition* × *modality* × *region*	2,34	0.159	0.761
*condition* × *hemisphere*	1,17	0.258	0.618
*modality* × *hemisphere*	1,17	0.008	0.932
*condition* × *modality* × *hemisphere*	1,17	0.041	0.842
*condition* × *region* × *hemisphere*	2,34	0.956	0.372
*modality* × *region* × *hemisphere*	2,34	0.288	0.751
*condition* × *modality* × *region* × *hemisphere*	2,34	1.553	0.226

**Table 2:** Statistical results of the ANOVA *condition* × *modality* × *region* × *hemisphere* for ERP data on midline electrodes. Data from the time window 500–900 ms was considered for spoken sentences, while data from the time window 800–1200 ms entered analyses for sung sentences. Significant effects (*p* < 0.050) are marked in bold.

*Effect Midline Electrodes*	*df*	*F*	*p*
***condition***	1,16	14.749	**0.001**
***modality***	1,16	8.033	**0.012**
*condition* × *modality*	1,16	0.191	0.668
***condition* × *electrodes***	2,32	5.428	**0.009**
*modality* × *electrodes*	2,32	3.142	0.057
*condition* × *modality* × *electrodes*	2,32	0.159	0.745

**Table 3:** Statistical results of the ANOVA *condition* × *modality* × *region* × *hemisphere* for [oxy-Hb] of functional near-infrared spectroscopy (fNIRS) data. Significant effects (*p* < 0.050) are marked in bold.

*Effect [oxy-Hb]*	*df*	*F*	*p*
*condition*	1,17	2.100	0.166
***modality***	1,17	4.897	**0.041**
*condition* × *modality*	1,17	0.041	0.841
*condition* × *region*	6,102	0.392	0.592
***modality* × *region***	6,102	3.211	**0.046**
*condition* × *modality* × *region*	6,102	0.614	0.624
*condition* × *hemisphere*	1,17	1.249	0.279
*modality* × *hemisphere*	1,17	1.012	0.329
*condition* × *modality* × *hemisphere*	1,17	0.018	0.896
*condition* × *region* × *hemisphere*	6,102	1.025	0.391
***modality* × *region* × *hemisphere***	6,102	4.165	**0.008**
*condition* × *modality* × *region* × *hemisphere*	6,102	1.615	0.198

**Table 4:** Statistical results of the ANOVA *condition* × *modality* × *region* × *hemisphere* for [deoxy-Hb] of fNIRS data. Significant effects (*p* < 0.50) are marked in bold.

*Effect [deoxy-Hb]*	*df*	*F*	*p*
***condition***	1,17	12.530	**0.003**
*modality*	1,17	1.153	0.298
*condition* × *modality*	1,17	0.020	0.889
*condition* × *region*	6,102	1.183	0.316
*modality* × *region*	6,102	0.936	0.416
*condition* × *modality* × *region*	6,102	1.555	0.210
*condition* × *hemisphere*	1,17	0.330	0.572
*modality* × *hemisphere*	1,17	0.675	0.423
*condition* × *modality* × *hemisphere*	1,17	1.573	0.227
*condition* × *region* × *hemisphere*	6,102	0.849	0.405
*modality* × *region* × *hemisphere*	6,102	0.669	0.520
*condition* × *modality* × *region* × *hemisphere*	6,102	0.532	0.648

## Supplementary Materials

The following are available online at https://www.mdpi.com/2076-3425/10/1/36/s1, Audio file S1: An example of a correct spoken sentence. Audio file S2: An example of a correct sung sentence. Audio file S3: An example of an incorrect spoken sentence. Audio file S4: An example of an incorrect sung sentence. Figure S5: Grand averages at the electrode Cz for semantically correct versus incorrect sentences for spoken and sung sentences aligned after the prefix “ge” of the critical past participle.

## References

[1] Kraus N., Slater J., Aminoff M.J., Boller F., Swaab D.F. (2015). Chapter 12-Music and language: Relations and disconnections. Handbook of Clinical Neurology.

[2] Seidner W., Wendler J. (1978). Die Sängerstimme.

[3] Sundberg J. (1970). Formant Structure and Articulation of Spoken and Sung Vowels. FPL (Folia Phoniatrica et Logopaedica).

[4] Zatorre R.J., Baum S.R. (2012). Musical Melody and Speech Intonation: Singing a Different Tune. PLoS Biol..

[5] Mithen S., Morley I., Wray A., Tallerman M., Gamble C. (2006). *The Singing Neanderthals: The Origins of Music, Language, Mind and Body, by Steven Mithen*; Weidenfeld & Nicholson: London, UK, 2005; pp. 97–112, ISBN 0-297-64317-7 hardback £20 & US$25.2; ix+374. Camb. Archaeol. J..

[6] De l’Etoile S.K. (2006). Infant behavioral responses to infant-directed singing and other maternal interactions. Infant Behav. Dev..

[7] Nakata T., Trehub S.E. (2004). Infants’ responsiveness to maternal speech and singing. Infant Behav. Dev..

[8] Crew J.D., Galvin J.J., Fu Q.-J. (2016). Perception of Sung Speech in Bimodal Cochlear Implant Users. Trends Hear..

[9] Yu M., Xu M., Li X., Chen Z., Song Y., Liu J. (2017). The shared neural basis of music and language. Neuroscience.

[10] Ludke K.M., Ferreira F., Overy K. (2014). Singing can facilitate foreign language learning. Mem. Cogn..

[11] Dittinger E., Barbaroux M., D’Imperio M., Jäncke L., Elmer S., Besson M. (2016). Professional Music Training and Novel Word Learning: From Faster Semantic Encoding to Longer-lasting Word Representations. J. Cogn. Neurosci..

[12] Kraus N., Chandrasekaran B. (2010). Music training for the development of auditory skills. Nat. Rev. Neurosci..

[13] Anderson S., White-Schwoch T., Parbery-Clark A., Kraus N. (2013). A dynamic auditory-cognitive system supports speech-in-noise perception in older adults. Hear. Res..

[14] Strait D.L., Kraus N. (2014). Biological impact of auditory expertise across the life span: Musicians as a model of auditory learning. Hear. Res..

[15] Schlaug G., Marchina S., Norton A. (2008). From Singing to Speaking: Why Singing May Lead to Recovery of Expressive Language Function in Patients with Broca’s Aphasia. Music Percept. Interdiscip. J..

[16] Merrett D.L., Peretz I., Wilson S.J. (2014). Neurobiological, Cognitive, and Emotional Mechanisms in Melodic Intonation Therapy. Front. Hum. Neurosci..

[17] Sihvonen A.J., Särkämö T., Leo V., Tervaniemi M., Altenmüller E., Soinila S. (2017). Music-based interventions in neurological rehabilitation. Lancet Neurol..

[18] Orellana C.P., van de Sandt-Koenderman M.E., Saliasi E., van der Meulen I., Klip S., van der Lugt A., Smits M. (2014). Insight into the neurophysiological processes of melodically intoned language with functional MRI. Brain Behav..

[19] Akanuma K., Meguro K., Satoh M., Tashiro M., Itoh M. (2016). Singing can improve speech function in aphasics associated with intact right basal ganglia and preserve right temporal glucose metabolism: Implications for singing therapy indication. Int. J. Neurosci..

[20] Good A., Gordon K.A., Papsin B.C., Nespoli G., Hopyan T., Peretz I., Russo F.A. (2017). Benefits of Music Training for Perception of Emotional Speech Prosody in Deaf Children With Cochlear Implants. Ear Hear..

[21] Torppa R., Faulkner A., Laasonen M., Lipsanen J., Sammler D. (2019). Links of Prosodic Stress Perception and Musical Activities to Language Skills of Children With Cochlear Implants and Normal Hearing. Ear Hear..

[22] Torppa R., Huotilainen M. (2019). Why and how music can be used to rehabilitate and develop speech and language skills in hearing-impaired children. Hear. Res..

[23] Rossi S., Telkemeyer S., Wartenburger I., Obrig H. (2012). Shedding light on words and sentences: Near-infrared spectroscopy in language research. Brain Lang..

[24] Holcomb P.J. (1993). Semantic priming and stimulus degradation: Implications for the role of the N400 in language processing. Psychophysiology.

[25] Rugg M.D. (1985). The Effects of Semantic Priming and Word Repetition on Event-Related Potentials. Psychophysiology.

[26] Matsumoto A., Iidaka T., Haneda K., Okada T., Sadato N. (2005). Linking semantic priming effect in functional MRI and event-related potentials. NeuroImage.

[27] Lau E.F., Phillips C., Poeppel D. (2008). A cortical network for semantics: (de)constructing the N400. Nat. Rev. Neurosci..

[28] Kutas M., Federmeier K.D. (2011). Thirty Years and Counting: Finding Meaning in the N400 Component of the Event-Related Brain Potential (ERP). Annu. Rev. Psychol..

[29] Koelsch S. (2011). Towards a neural basis of processing musical semantics. Phys. Life Rev..

[30] Koelsch S. (2011). Toward a Neural Basis of Music Perception-A Review and Updated Model. Front. Psychol..

[31] Koelsch S., Kasper E., Sammler D., Schulze K., Gunter T., Friederici A.D. (2004). Music, language and meaning: Brain signatures of semantic processing. Nat. Neurosci..

[32] Steinbeis N., Koelsch S. (2008). Comparing the Processing of Music and Language Meaning Using EEG and fMRI Provides Evidence for Similar and Distinct Neural Representations. PLoS ONE.

[33] Painter J.G., Koelsch S. (2011). Can out-of-context musical sounds convey meaning? An ERP study on the processing of meaning in music: Processing of meaning in music. Psychophysiology.

[34] Chien P.-J., Chan S. (2015). Old songs can be as fresh as new: An ERP study on lyrics processing. J. Neurolinguist..

[35] Gordon R.L., Schön D., Magne C., Astésano C., Besson M. (2010). Words and Melody Are Intertwined in Perception of Sung Words: EEG and Behavioral Evidence. PLoS ONE.

[36] Kutas M., Hillyard S.A. (1980). Reading senseless sentences: Brain potentials reflect semantic incongruity. Science.

[37] Friederici A.D. (2002). Towards a neural basis of auditory sentence processing. Trends Cogn. Sci. (Regul. Ed.).

[38] Hahne A., Friederici A.D. (2002). Differential task effects on semantic and syntactic processes as revealed by ERPs. Cogn. Brain Res..

[39] Friederici A.D., Pfeifer E., Hahne A. (1993). Event-related brain potentials during natural speech processing: Effects of semantic, morphological and syntactic violations. Cogn. Brain Res..

[40] Besson M., Faïta F., Peretz I., Bonnel A.-M., Requin J. (1998). Singing in the Brain: Independence of Lyrics and Tunes. Psychol. Sci..

[41] Rosslau K., Herholz S.C., Knief A., Ortmann M., Deuster D., Schmidt C.-M., Zehnhoff-Dinnesen A., Pantev C., Dobel C. (2016). Song Perception by Professional Singers and Actors: An MEG Study. PLoS ONE.

[42] Besson M., Schön D. (2001). Comparison between Language and Music. Ann. N. Y. Acad. Sci..

[43] Zatorre R.J., Belin P., Penhune V.B. (2002). Structure and function of auditory cortex: Music and speech. Trends Cogn. Sci..

[44] Zatorre R.J., Belin P. (2001). Spectral and Temporal Processing in Human Auditory Cortex. Cereb. Cortex.

[45] Poeppel D., Idsardi W.J., van Wassenhove V. (2008). Speech perception at the interface of neurobiology and linguistics. Philos. Trans. R. Soc. B Biol. Sci..

[46] Telkemeyer S., Rossi S., Koch S.P., Nierhaus T., Steinbrink J., Poeppel D., Obrig H., Wartenburger I. (2009). Sensitivity of Newborn Auditory Cortex to the Temporal Structure of Sounds. J. Neurosci..

[47] Friederici A.D., Alter K. (2004). Lateralization of auditory language functions: A dynamic dual pathway model. Brain Lang..

[48] Hickok G., Poeppel D. (2007). The cortical organization of speech processing. Nat. Rev. Neurosci..

[49] Mummery C.J., Shallice T., Price C.J. (1999). Dual-Process Model in Semantic Priming: A Functional Imaging Perspective. NeuroImage.

[50] Fang Y., Han Z., Zhong S., Gong G., Song L., Liu F., Huang R., Du X., Sun R., Wang Q. The Semantic Anatomical Network: Evidence from Healthy and Brain-Damaged Patient Populations. https://onlinelibrary.wiley.com/doi/abs/10.1002/hbm.22858.

[51] Rissman J., Eliassen J.C., Blumstein S.E. (2003). An event-related FMRI investigation of implicit semantic priming. J. Cogn. Neurosci..

[52] Sammler D., Baird A., Valabrègue R., Clément S., Dupont S., Belin P., Samson S. (2010). The Relationship of Lyrics and Tunes in the Processing of Unfamiliar Songs: A Functional Magnetic Resonance Adaptation Study. J. Neurosci..

[53] Schön D., Gordon R., Campagne A., Magne C., Astésano C., Anton J.-L., Besson M. (2010). Similar cerebral networks in language, music and song perception. NeuroImage.

[54] Merrill J., Sammler D., Bangert M., Goldhahn D., Lohmann G., Turner R., Friederici A.D. (2012). Perception of Words and Pitch Patterns in Song and Speech. Front. Psychol..

[55] Kreitewolf J., Friederici A.D., von Kriegstein K. (2014). Hemispheric lateralization of linguistic prosody recognition in comparison to speech and speaker recognition. NeuroImage.

[56] Callan D.E., Tsytsarev V., Hanakawa T., Callan A.M., Katsuhara M., Fukuyama H., Turner R. (2006). Song and speech: Brain regions involved with perception and covert production. NeuroImage.

[57] Whitehead J.C., Armony J.L. (2018). Singing in the brain: Neural representation of music and voice as revealed by fMRI. Hum. Brain Mapp..

[58] Özdemir E., Norton A., Schlaug G. (2006). Shared and distinct neural correlates of singing and speaking. NeuroImage.

[59] Humphries C., Binder J.R., Medler D.A., Liebenthal E. (2006). Syntactic and Semantic Modulation of Neural Activity during Auditory Sentence Comprehension. J. Cogn. Neurosci..

[60] Kuperberg G.R., McGuire P.K., Bullmore E.T., Brammer M.J., Rabe-Hesketh S., Wright I.C., Lythgoe D.J., Williams S.C.R., David A.S. (2000). Common and Distinct Neural Substrates for Pragmatic, Semantic, and Syntactic Processing of Spoken Sentences: An fMRI Study. J. Cogn. Neurosci..

[61] Rüschemeyer S.-A., Fiebach C.J., Kempe V., Friederici A.D. Processing Lexical Semantic and Syntactic Information in First and Second Language: fMRI Evidence from German and Russian. https://onlinelibrary.wiley.com/doi/abs/10.1002/hbm.20098.

[62] Friederici A.D., Rüschemeyer S.-A., Hahne A., Fiebach C.J. (2003). The Role of Left Inferior Frontal and Superior Temporal Cortex in Sentence Comprehension: Localizing Syntactic and Semantic Processes. Cereb. Cortex.

[63] Rogalsky C., Hickok G. (2009). Selective Attention to Semantic and Syntactic Features Modulates Sentence Processing Networks in Anterior Temporal Cortex. Cereb. Cortex.

[64] Oldfield R.C. (1971). The assessment and analysis of handedness: The Edinburgh inventory. Neuropsychologia.

[65] Huettel S.A., Song A.W., McCarthy G. (2008). Functional Magnetic Resonance Imaging.

[66] Benjamini Y., Hochberg Y. (1995). Controlling the False Discovery Rate: A Practical and Powerful Approach to Multiple Testing. J. R. Stat. Soc. Ser. B (Methodological).

[67] Gratton G., Coles M.G., Donchin E. (1983). A new method for off-line removal of ocular artifact. Electroencephalogr. Clin. Neurophysiol..

[68] Greenhouse S.W., Geisser S. (1959). On methods in the analysis of profile data. Psychometrika.

[69] Scholkmann F., Spichtig S., Muehlemann T., Wolf M. (2010). How to detect and reduce movement artifacts in near-infrared imaging using moving standard deviation and spline interpolation. Physiol. Meas..

[70] Cope M., Delpy D.T., Wray S., Wyatt J.S., Reynolds E.O.R. (1989). A CCD Spectrophotometer to Quantitate the Concentration of Chromophores in Living Tissue Utilising the Absorption Peak of Water at 975 nm. Oxygen Transport to Tissue XI.

[71] Boynton G.M., Engel S.A., Heeger D.J. (2012). Linear systems analysis of the fMRI signal. NeuroImage.

[72] Cheimariou S., Farmer T.A., Gordon J.K. (2019). Lexical prediction in the aging brain: The effects of predictiveness and congruency on the N400 ERP component. Aging Neuropsychol. Cogn..

[73] Kutas M., Iragui V. (1998). The N400 in a semantic categorization task across 6 decades. Electroencephalogr. Clin. Neurophysiol. Evoked Potentials Sect..

[74] Hunter C.R. (2016). Is the time course of lexical activation and competition in spoken word recognition affected by adult aging? An event-related potential (ERP) study. Neuropsychologia.

[75] Mohan R., Weber C. (2019). Neural activity reveals effects of aging on inhibitory processes during word retrieval. Aging Neuropsychol. Cogn..

[76] Federmeier K.D., Van Petten C., Schwartz T.J., Kutas M. (2003). Sounds, Words, Sentences: Age-Related Changes Across Levels of Language Processing. Psychol. Aging.

[77] Friederici A.D., von Cramon D.Y., Kotz S.A. (1999). Language related brain potentials in patients with cortical and subcortical left hemisphere lesions. Brain.

[78] Hagoort P., Brown C.M., Swaab T.Y. (1996). Lexical-semantic event–related potential effects in patients with left hemisphere lesions and aphasia, and patients with right hemisphere lesions without aphasia. Brain.

[79] Swaab T., Brown C., Hagoort P. (1997). Spoken Sentence Comprehension in Aphasia: Event-related Potential Evidence for a Lexical Integration Deficit. J. Cogn. Neurosci..

