# Supplementary figures and images for: How the Brain Understands Spoken and Sung Sentences
Source: Brain Sci. 2020 Jan 8;10(1):36. doi: 10.3390/brainsci10010036 (PMC7017195; doi:10.3390/brainsci10010036)

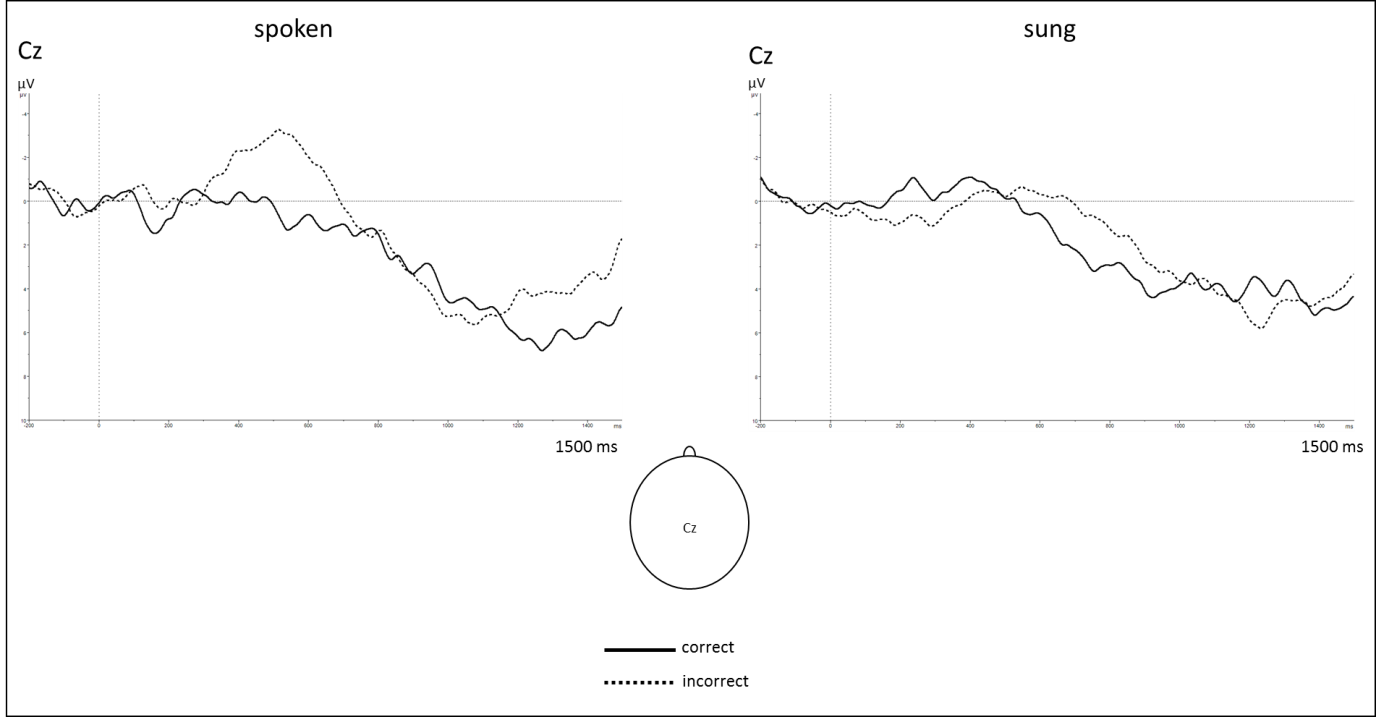

Supplement: Supplementary file 1 [file brainsci-10-00036-s001.zip › s5_figure.png]
